# Monitoring of pesticides water pollution-The Egyptian River Nile

**DOI:** 10.1186/s40201-016-0259-6

**Published:** 2016-10-07

**Authors:** Hesham Dahshan, Ayman Mohamed Megahed, Amr Mohamed Mohamed Abd-Elall, Mahdy Abdel-Goad Abd-El-Kader, Ehab Nabawy, Mariam Hassan Elbana

**Affiliations:** Department of Veterinary Public Health, Faculty of Veterinary Medicine, Zagazig University, Zagazig, Sharkia governorate Egypt

**Keywords:** Monitoring, Organochlorine pesticides, Organophosphorus pesticides, River Nile, Human hazardous risk

## Abstract

**Background:**

Persistent organic pollutants represent about 95 % of the industrial sector effluents in Egypt. Contamination of the River Nile water with various pesticides poses a hazardous risk to both human and environmental compartments. Therefore, a large scale monitoring study was carried on pesticides pollution in three geographical main regions along the River Nil water stream, Egypt.

**Methods:**

Organochlorine and organophosphorus pesticides were extracted by liquid-liquid extraction and analyzed by GC-ECD.

**Results:**

Organochlorine pesticides mean concentrations along the River Nile water samples were 0.403, 1.081, 1.209, 3.22, and 1.192 μg L^−1^ for endrin, dieldrin, p, p’-DDD, p, p’-DDT, and p, p’-DDE, respectively. Dieldrin, p, p’-DDT, and p, p’-DDE were above the standard guidelines of the World Health Organization. Detected organophosphorus pesticides were Triazophos (2.601 μg L^−1^), Quinalphos (1.91 μg L^−1^), fenitrothion (1.222 μg L^−1^), Ethoprophos (1.076 μg L^−1^), chlorpyrifos (0.578 μg L^−1^), ethion (0.263 μg L^−1^), Fenamiphos (0.111 μg L^−1^), and pirimiphos-methyl (0.04 μg L^−1^). Toxicity characterization of organophosphorus pesticides according to water quality guidelines indicated the hazardous risk of detected chemicals to the public and to the different environmental compartments. The spatial distribution patterns of detected pesticides reflected the reverse relationship between regional temperature and organochlorine pesticides distribution. However, organophosphorus was distributed according to the local inputs of pollutant compounds.

**Conclusions:**

Toxicological and water quality standards data revealed the hazardous risk of detected pesticides in the Egyptian River Nile water to human and aquatic life. Thus, our monitoring data will provide viewpoints by which stricter legislation and regulatory controls can be admitted to avoid River Nile pesticide water pollution.

## Background

Over fifty years pesticides were used in African countries for combating and controlling agricultural pests [1]. Among African countries, Egypt is one of the intensive pesticide use areas. Thus, the main water supply (River Nile) is loaded with various types of persistent organic pollutants (POPs).

Nowadays, POPs represent about 95 % of the major industrial sectors in Egypt as raw and fabricated metals, vehicles, pharmaceuticals, textiles, pesticides, fertilizers, petrochemicals, cement, paper and pulp, and food processing [2].

Organochlorine pesticides represent an important group of POPs, which are believed to be possible carcinogens as well as endocrine disruptors [3]. Due to its hazardous risk, the United Nations Environmental Program (UNEP) has initiated a prospective for reducing these threatening chemicals worldwide as agricultural sectors have forced to be shifted towards organophosphorus pesticides instead of organochlorine. However, these compounds are more toxic to vertebrates than other classes of insecticides [4].

In Egypt, the River Nile ecosystem is of particular interest since river water is the main source of drinking for about 90 million citizens and also the main way for irrigation of agricultural lands. The monitoring and assessment of pesticide water pollution have been well studied in North America, Japan and many Europe countries [5]. However, studies on freshwater aquatic environments in Egypt are scanty especially the large-scale monitoring studies. A former study was carried in 1995 on freshwater aquatic environments along the River Nile revealed that DDT, HCH, and PCBs were detected [6]. Furthermore, inconstancy of organochlorine residues in Nile water during the period from 1982 to 1998 was reported in literatures [7, 8]. For our point of view, no data about the River Nile pesticides water pollution is available since 1998.

As monitoring of pesticide water pollution is an substantial source of information describing the current state of environmental pollution and reflecting the effectiveness of environmental legislation policies, our study was conducted to obtain a large scale monitoring data on spatial distribution of selected organochlorine and organophosphorus pesticides in water samples collected at 20 sampling sites along the River Nile stream and the major delta lakes of Egypt.

## Methods

### Study area

**Fig. 1:**
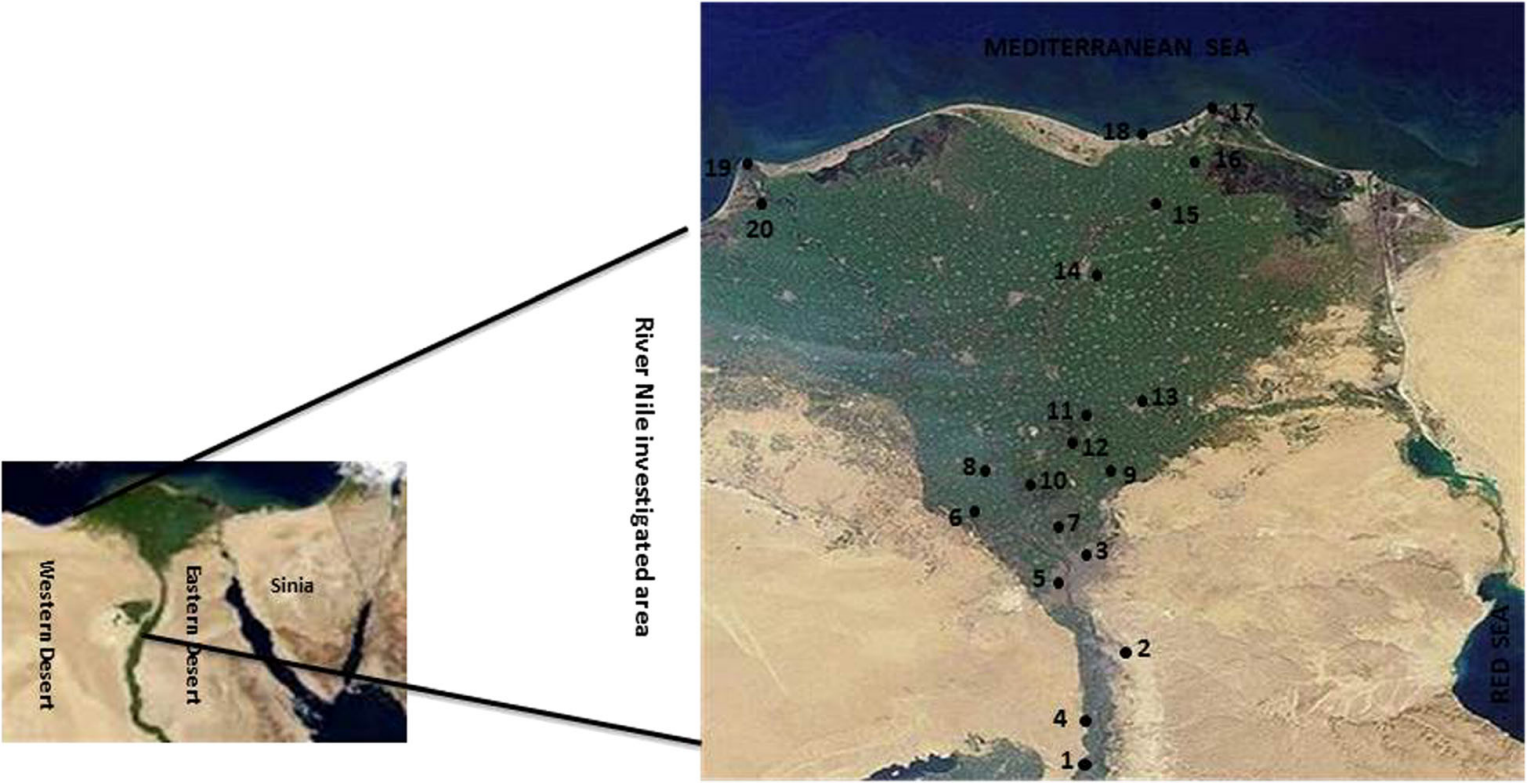
Map of the River Nile showing sites of water sampling: Greater Cairo; 1. Alwasta-Beni Sweif, 2. Helwan, 3. Cairo, 4. Alaeat-Giza, 5. Giza town, 6. Alknater-Giza, 7. Qalubiya& Nile Delta; 8. Monofea, 9. Belbas, 10. Benha, 11. Alazezea-Menya Elkamh, 12. Menya Elkamh town, 13. Zagazig, 14. Mansoura& Nile estuaries; 15. Fraskour-Damietta, 16. Damietta town, 17. Ras El-bar, 18. Gamasa, 19. Rosetta town, 20. Edfina-Rosetta

A large-scale monitoring study was conducted on organochlorine and organophosphorus residue levels in water samples collected at 20 sampling sites along the River Nile, Egypt (Fig. 1). Sampling regions were selected according to the locations of major agricultural and industrial activities. Three geographical regions along the River Nile stream were selected as follows; Greater Cairo, in which about 50 % of all industrial activity is concentrated & Nile Delta, the majority of agricultural lands are located and the remaining industrial activity rests (vehicles, textiles, fertilizers, food, detergents) and & Nile estuaries at Damietta and Rosetta, in which textiles, furniture, pesticides, food factories are the main national income. From Greater Cairo, seven sampling sites were selected. Meanwhile, from Nile Delta, Nile estuaries seven and six sampling sites were selected, respectively.

### Sample collection

During the summer of 2013, a total of 60 water sample were collected from the River Nile sampling sites (3 samples each). Water samples were collected using 2.5 L amber glass bottle at 50 cm below water surface. Water samples were filtered through 0.45 μm fiber glass filters to remove sand and debris (WHATMAN) [9, 10].

### Reagents and standards

Reagents used are; solvents including, n-hexane, acetonitrile, ethanol, and dichloromethane (all solvents were pesticide residue (PR) grade and were purchased from Alliance Bio, USA. Alliance Bio, USA); florisil 60–100 mesh (Sigma, USA); and sodium sulfate anhydrate (El Nasr Pharmaceutical Chemical Co, Egypt). The individual reference standards used for quantification and identification of organochlorine and organophosphorus residues were obtained from Dr. Ehrenstorfer GmbH (Augsburg, Germany).

### Analytical procedures

#### Extraction

Liquid-liquid extraction was used according to procedures described by APHA [11]. Water sample was extracted twice. In each, A 60 mL volume of 15 % methylene chloride in n-hexane was introduced into a 2 L separating funnel containing 1 L of filtered water and shaken vigorously for 5 min. The combined extracts were dried over anhydrous sodium sulfate and concentrated to about 1 mL in a rotary evaporator.

#### Clean up

Water extract were cleaned and fractionated on florisil; 20 g of 0.5 % activated florisil was poured into a column topped with 1 g anhydrous sodium sulfate to remove remaining water from the sample. The florisil column was washed with 50 mL n-hexane before the sample loaded. To recover p, p’-DDE, the column was eluted with 60 mL of 30 % methylene chloride in n-hexane (First fraction). The second fraction was achieved by column elution with 35 mL of 30 % dichloromethane in hexane and after that with 45 mL of 50 % dichloromethane in hexane to elute all organochlorine pesticide residues in the samples. Each fraction was evaporated in the rotary vacuum evaporator until the volume reached 2–3 mL [12, 13]. However, to determine the residues of organophosphorus pesticide, the water sample extract was injected in gas chromatography for analysis without clean up.

#### Quantitative determination

Quantitative analysis of pesticides was carried out at Residue Analysis Department, Central Agri. Pesticides Lab, Dokki, Egypt using an Agilent gas chromatograph 6890 coupled with a HP-5MS (Agilent, Folsom, CA) capillary column of 30 m length × 0.25 mm internal diameter × 0.25 μm film thickness, Agilent). Chemstation software was used for instrument control. A 63Ni-ECD detector was used for analysis. The GC system was operated in a splitless mode. The column oven temperature was programmed as follows; the oven temperature was programmed from an initial temperature 180 °C (2 min hold) to 220 °C (1 min hold) at a rate of 5 °C/min, then finally to 280 °C at a rate of 9 °C/min. the oven was maintained at 280 °C for 30 min. The temperature of the injector operating in splitless mode was held at 260 °C while the detector temperature was 320 °C. The carrier gas was ultra pure nitrogen at flow rate of 4 mL/min. The target compounds were identified on the basis of the retention times of individual authentic standards.

#### Quality assurance and quality control

The quality of organochlorine and organophosphorus pesticides was assured through the analysis of solvent blanks, procedure blanks and triplicate samples. LOD and LOQ data in the GC-ECD was presented in Table 1. Sample of each series was analyzed in triplicates.

**Table 1:** Analyzed pesticides, limits of detection and limits of quantification data in the GC-ECD (μg/L^−1^)

Pesticides	LOD	LOQ
α-HCH	0.005	0.015
γ-HCH	0.004	0.012
Aldrin	0.003	0.009
Heptachlor	0.003	0.009
Endrin	0.003	0.009
Heptachlor epoxide	0.003	0.009
P, P’-DDE	0.003	0.009
Dieldrin	0.002	0.006
P, P’-DDD	0.003	0.009
P, P’-DDT	0.004	0.012
Ethoprophos	0.005	0.015
Phorate Diazinon	0.003	0.009
Dimethoate	0.005	0.015
Pirimiphos-methyl	0.005	0.015
Chlorpyrifos	0.005	0.015
Fenitrothion	0.004	0.012
Quinalphos	0.005	0.015
Prothiofos Ethion	0.005	0.015
Triazophos	0.004	0.012
Fenamiphos	0.005	0.015

## Results and discussion

### Pollution of the River Nile water by pesticides

The quantities of pesticides used in Egypt based on Environmental Affairs agency, Egypt; January 2009, is about 600 ton/annually. Therefore, the spatial distribution of ten organochlorine and twelve organophosphorus pesticide residues in the main water source for Egyptian (River Nile) was investigated.

#### Occurrence of organochlorine pesticide residues

**Fig. 2:**
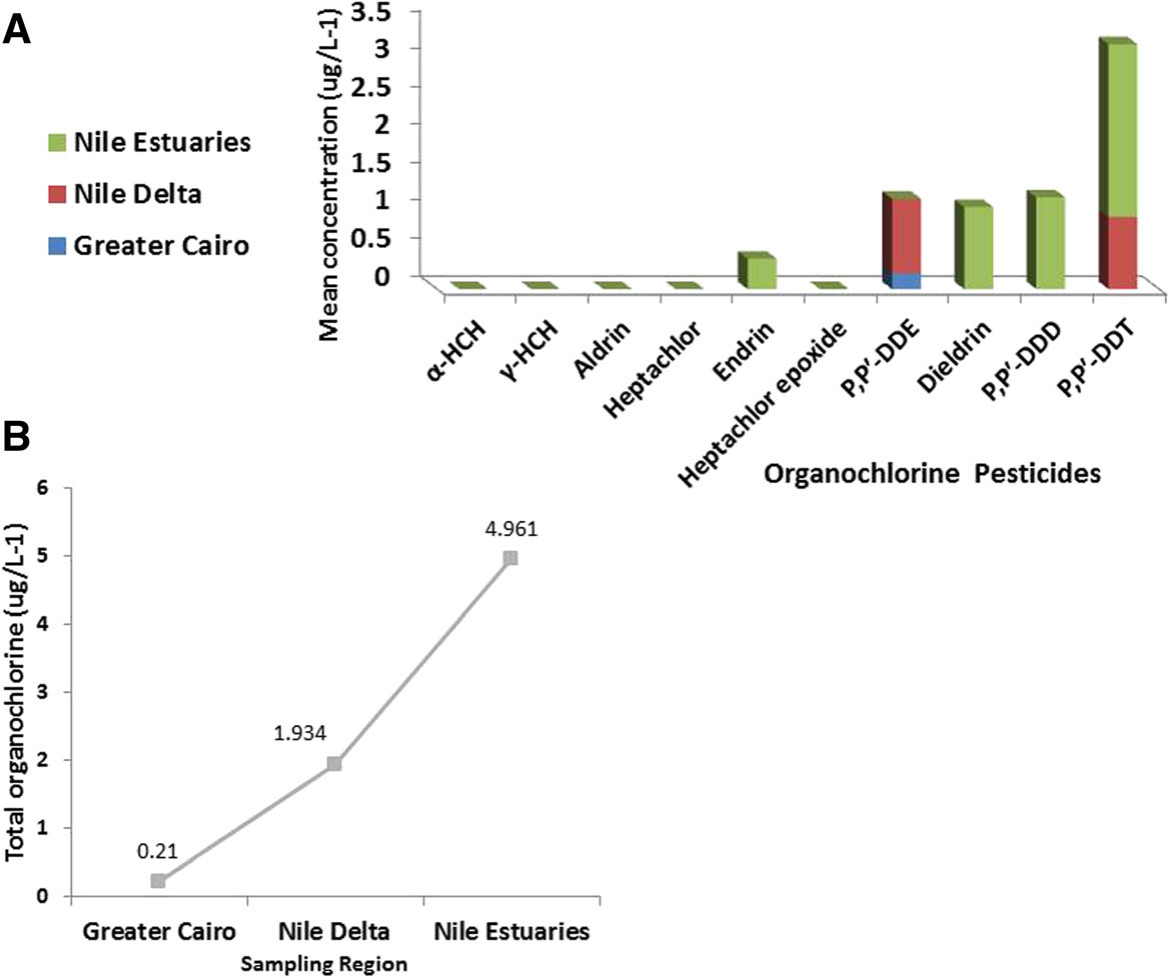
**a** Mean organochlorine pesticide concentrations; **b** Spatial distribution of total organochlorine pesticides in water samples collected from three sampling regions along the River Nile, Egypt

Water samples taken from three studied regions (Greater Cairo, Nile Delta, Nile estuaries at Damietta and Rosetta) at River Nile were analyzed. The recoveries of organochlorine pesticides ranged between 82 and 98.6 %. The mean concentration values are presented in Table 2. Organochlorine pesticide residues were mainly detected in the downstream of the river as follows; endrin, dieldrin, p, p’-DDD, and p, p’-DDT at a rate of 0.403, 1.081, 1.209, and 2.268 μg L^−1^, respectively. The levels of DDTs in this study were higher than those in the Pearl River, the Haihe River, Qiantang River and the Huaihe River [14–17]. However, the concentration is lower than the concentration obtained from water sample collected in Begumganj, Bangladesh [18]. The high concentration level of total organochlorine pesticides at the Nile estuaries (Fig. 2b) could be attributed to the Delta agricultural lands wash off. Further investigations are clearly needed to reveal the sources and patterns of organochlorine pesticides contamination in river water.

**Table 2:** Mean concentration of organochlorine pesticides (μg/L^−1^) detected in water samples from the River Nile, Egypt

Site No.	Site Name	Region	α-HCH	γ-HCH	Aldrin	Heptachlor	Endrin (2 μg/L^−1^)^a^	Heptachlor epoxide	P, P’-DDE (2 μg/L^−1^)^a^	Dieldrin (0.03 μg/L^−1^)^a^	P, P’-DDD (2 μg/L^−1^)^a^	P, P’-DDT (2 μg/L^−1^)^a^	Total organochlorine pesticides
1	Alwasta-Beni Sweif	Greater Cairo	ND	ND	ND	ND	ND	ND	0.21	ND	ND	ND	0.21
2	Helwan												
3	Cairo												
4	Alaeat-Giza												
5	Giza town												
6	Alknater-Giza												
7	Qalubiya												
8	Monofea	Nile Delta	ND	ND	ND	ND	ND	ND	0.982	ND	ND	0.952	1.934
9	Belbas												
10	Benha												
11	Alazezea-Menya Elkamh												
12	Menya Elkamh town												
13	Zagazig												
14	Mansoura												
15	Fraskour-Damietta	Nile Estuaries	ND	ND	ND	ND	0.403	ND	ND	**1.081**	1.209	**2.268**	4.961
16	Damietta town												
17	Ras El-bar												
18	Gamasa												
19	Rosetta town												
20	Edfina-Rosetta												
													7.105^a^

Bold numbers: Values above the standard guidelines of World Health Organization

^a^Organochlorine pesticide concentration (μg/L^−1^) along the River Nile sampling sites

*ND* not detectable, Number of samples = 60 (3/each sampling site)

It is surprising to note that at Greater Cairo and Nile Delta region, various organochlorine pesticides were not detected although major industrial and agricultural activities are concentrated there. This could be due to the pesticides evaporation in tropical countries (Egypt), pesticide residues dilution or adsorption. p, p’-DDE was detected only at Greater Cairo in a low concentration, (0.21 μg L^−1^). However at Nile Delta region, p, p’-DDE and p, p’-DDT was estimated in a concentration of 0.982 and 0.952 μg L^−1^, respectively Table 2.

Along the investigated River Nile region sites, the most frequently detected organochlorine pesticide was endrin. Followed by, dieldrin, p, p’-DDE, p, p’-DDD, and p, p’-DDT. However, α-HCH, γ-HCH, aldrin, heptachlor, and heptachlor epoxide were not detected in the water samples (Fig. 2a). In spite of, p, p’-DDT and its metabolites (p, p’-DDE and p, p’-DDD), endrin and dieldrin have been officially prohibited since 1980 and in 1996 a Ministerial Decree prohibited the import and use of 80 pesticides including dieldrin, endrin, and DDT [19]. Nonetheless, our study indicates that above mentioned organochlorine pesticides are still sold in Egyptian markets.

#### Occurrence of organophosphorus pesticide residues

**Fig. 3:**
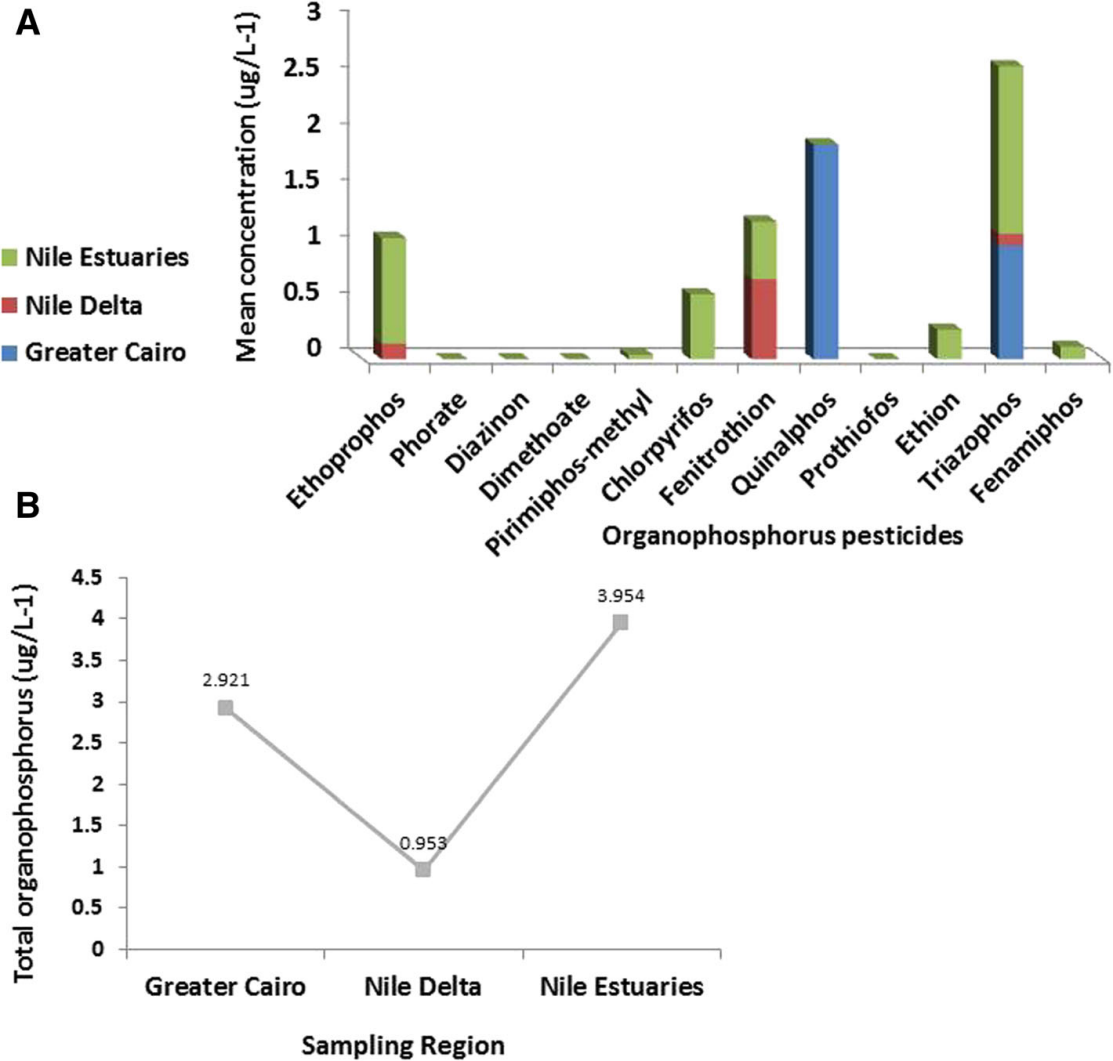
**a** Mean organophosphorus pesticide concentrations; **b** Spatial distribution of organophosphorus pesticides in water samples collected from three sampling regions along the River Nile, Egypt

Amongst 12 organophosphorus pesticides analyzed, eight were detected. The recoveries of organophosphorus pesticides were in-between 82.5 and 100 %. The most frequently detected was triazophos, followed by quinalphos, then, fenitrothion, ethoprophos, chlorpyrifos, ethion, fenamiphos, and pirimiphos-methyl. However, prothiofos, dimethoate, diazinon, and phorate were not detected (Fig. 3a). For the Nile estuaries, the highest concentration of organophosphorus pesticide detected in water was 1.488 μg L^−1^ for triazophos. In our monitoring study levels of triazophos are generally higher than those reported in rivers and lakes of Greece [20], River Ravi of Pakistan [21], potable and irrigated water of Brazil [22]. Our results are in concert with a study conducted in Jiulong River in South China [23] as triazophos was the main organophosphorus pesticides detected in the estuary river water. In general, studying of organophosphorus River Nile water pollution is still in its initial stage, and further research is increasingly needed to establish a frame network data about its contamination degree.

In Greater Cairo and Nile Delta sampling regions the higher concentrations were 1.91 and 0.711 μg L^−1^ for, quinalphos, and fenitrothion, respectively Table 3. Accordingly, our results revealed that organophosphorus pesticide concentrations in the River Nile water, Egypt exceeded the EEC Council Directive 98/83/EC for water quality standard [24]. This could be attributed to the substitution of persistent organochlorine pesticides with organophosphate pesticides in the treatment of scattered cotton fields in Egypt as organophosphates and carbamates are the dominate insecticide used there [25, 26], resulting in serious hazards to the freshwater aquatic environments and adverse harmful effects to wildlife and humans.

**Table 3:** Mean concentration of organophosphorus pesticides (μg/L^−1^) detected in water samples from the River Nile, Egypt

Site No.	Site Name	Region	Ethoprophos	Phorate	Diazinon	Dimethoate	Pirimiphos-methyl	Chlorpyrifos	Fenitrothion	Quinalphos	Prothiofos	Ethion	Triazophos	Fenamiphos	Total organophosphorus pesticides
1	Alwasta-Beni Sweif	Greater Cairo	ND	ND	ND	ND	ND	ND	ND	1.91	ND	ND	1.011	ND	2.921
2	Helwan														
3	Cairo														
4	Alaeat-Giza														
5	Giza town														
6	Alknater-Giza														
7	Qalubiya														
8	Monofea	Nile Delta	0.14	ND	ND	ND	ND	ND	0.711	ND	ND	ND	0.102	ND	0.953
9	Belbas														
10	Benha														
11	Alazezea-Menya Elkamh														
12	Menya Elkamh town														
13	Zagazig														
14	Mansoura														
15	Fraskour-Damietta	Nile Estuaries	0.936	ND	ND	ND	0.04	0.578	0.511	ND	ND	0.263	1.488	0.111	3.954
16	Damietta town														
17	Ras El-bar														
18	Gamasa														
19	Rosetta town														
20	Edfina-Rosetta														
															7.828^a^

^a^Organophosphorus pesticide concentration (μg/L^−1^) along the River Nile sampling sites

*ND* not detectable; Number of samples = 60 (3/each sampling site)

### Spatial distribution of pesticides in the River Nile water samples

#### Organochlorine pesticides

Organochlorine pesticides water pollution showed a gradual increase in total organochlorine concentrations from Nile upstream at Greater Cairo in which total organochlorine pesticides were 0.21 μg L^−1^ to the Nile estuaries in which total organochlorine pesticides were 4.961 μg L^−1^. (Fig. 2b). In this context, we can expect the reverse relationship between the spatial organochlorine pesticides distribution and sampling regions temperature as organochlorine pesticides volatilize at warm temperatures (Nile upstream) and condense at cooler temperatures, reaching their highest concentrations in the cooler regions (Nile estuaries) [27].

#### Organophosphorus pesticides

Residues of total organophosphorus pesticides along the River Nile water sampling regions, showing the following spatial distribution pattern: River Nile estuaries > Greater Cairo > Nile Delta (Fig. 3b). Each sampling region was highly contaminated by special organophosphorus compound (Fig. 3a). The Fluxes in organophosphorus levels along the River Nile indicate contaminants local inputs. No cumulative effect toward the river downstream as Greater Cairo water samples were more contaminated by organophosphorus pesticides than Nile Delta samples in spite of its geographical location toward the river upstream Fig. 1.

### Human hazardous risks

Human exposure to pesticide residues could be through water, food and air. Residue levels vary according to the type of exposure and the individual’s daily intake [28]. Therefore, the assessing of human hazardous risks due to the intake of pesticides polluted water is important.

#### Organochlorine pesticides

The hazardous risk of organochlorine pesticides was evaluated according to water quality guidelines set by the World Health Organization (WHO), which specifies limits for endrin, p, p’-DDE, dieldrin, p, p’-DDD, and p, p’-DDT as 2, 2, 0.03, 2, and 2 μg L^−1^, respectively [29]. Our results showed that dieldrin and p, p’-DDT residues in some sampling sites were above the standard guidelines of WHO Table 2. Thus, water from the River Nile generally possessed an environmental and human health hazard as dieldrin is highly toxic to the central nervous system [30] and eating DDT contaminated fish over a short time would most likely affect the nervous system [31].

#### Organophosphorus pesticides

Of the organophosphorus pesticides detected in water, Ethoprophos, Triazophos, and Fenamiphos are considered highly hazardous to fish and other aquatic organisms, while others are considered moderately to slightly toxic. Water quality standards and toxicological data for human and aquatic organisms in relation to the detected organophosphorus pesticides are listed in Table 4. Toxicity characterization based on the Pesticide Action Network databases, WHO, Canadian Water Quality Guidelines, and U.S. National Drinking Water Standards and Health Criteria, revealed that all the detected organophosphorus pesticides are related to at least one health effect [32]. Thus, new tools and policies with greater reliability than those already existing by the Egyptian Environmental Affairs Agency (EEAA) of the Ministry of State for Environmental Affairs are needed to prevent or reduce the use of these harmful chemicals in industrial and agricultural sectors.

**Table 4:** Hazardous risks of detected organophosphorus pesticides in the River Nile, Egypt

Detected Organophosphorus compound	Total Concentration along the River Nile μg/L^−1^	PAN Bad Actors^b^	WHO Acute Hazard	Carcinogen	WHO Water Quality Criteria μg/L^−1^	Canadian Water Quality Guidelines for the Protection of Aquatic Life μg/L^−1^
Ethoprophos	1.076	Yes	Ia, Extremely hazardous	Yes	No water quality standard.	No water quality guidelines but induce mortality.
Pirimiphos-methyl	0.04	Yes	III, Slightly hazardous	Unclassifiable	Not recommended for direct application to drinking water.	No water quality guidelines but (Moderate to high toxicity)
Chlorpyrifos	0.578	Yes	II, Moderately hazardous	Not likely	30.0	0.0035
Fenitrothion	1.222	Yes	II, Moderately hazardous	Not likely	Occurs at concentrations below toxic effects.	Moderately toxic
Quinalphos	1.91	Yes	II, Moderately hazardous	Not likely	No water quality standard.	No water quality guidelines but induce mortality.
Ethion	0.263	Yes	II, Moderately hazardous	Not likely	No water quality standard.	No water quality guidelines (Moderate to high toxicity)
Triazophos	2.601	Yes	Ib, Highly hazardous	Not likely	Unlikely to occur.	Unlikely to occur, but induce mortality.
Fenamiphos	0.111	Yes	Ib, Highly hazardous	Not likely	3.50^a^	Unlikely to occur, but induce mortality.

Data of Hazardous risk were presented according to (Kegley et al., 2014) [32]

^a^U.S. Drinking Water Equivalent Level cited by U.S. National Drinking Water Standards and Health Criteria

^b^Pan Bad Actors: are chemicals that are highly acutely toxic, cholinesterase inhibitor

## Conclusions

Organochlorine and organophosphorus pesticides River Nile water pollution was investigated. Organochlorine pesticides detected were dieldrin; endrin; p, p’-DDE; p, p’-DDD; and p, p’-DDT. While, organophosphorus pesticides detected were triazophos, ethoprophos, quinalphos, chlorpyrifos, fenitrothion, ethion, fenamiphos, and pirimiphos-methyl. Spatial distribution of detected pesticides showed the reverse relationship between sampling regions temperature and organochlorine pesticides distribution. Meanwhile, organophosphorus pesticides were distributed according to the local inputs of pollutant compounds. Toxicological and water quality standards data revealed the hazardous risk of detected chemicals to human and aquatic life. We expect our results will provide viewpoints by which stricter legislation and regulatory controls can be admitted to avoid River Nile pesticide water pollution.

## Abbreviations

μg L^−1^: Microgram/litter
APHA: American public health association
DDD: Dichlorodiphenyldichloroethane
DDE: Dichlorodiphenyldichloroethylene
DDT: Dichlorodiphenyltrichloroethane
ECD: Electron capture detector
EEAA: Egyptian environmental affairs agency
EECD: European economic community directive
GC: Gas chromatography
LOD: Limit of detection
LOQ: Limit of quantification
PCBs: Polychlorinated biphenyls
POPs: Persistent organic pollutants
UNEP: United Nations environmental program
WHO: World Health Organization
α-HCH: alpha-Hexachlorocyclohexane
γ-HCH: gamma-Hexachlorocyclohexane
